# Association and interaction effects of Alzheimer’s disease-associated genes and lifestyle on cognitive aging in older adults in a Taiwanese population

**DOI:** 10.18632/oncotarget.15269

**Published:** 2017-02-11

**Authors:** Eugene Lin, Shih-Jen Tsai, Po-Hsiu Kuo, Yu-Li Liu, Albert C. Yang, Chung-Feng Kao

**Affiliations:** ^1^ Institute of Biomedical Sciences, China Medical University, Taichung, Taiwan; ^2^ Vita Genomics, Inc., Taipei, Taiwan; ^3^ TickleFish Systems Corporation, Seattle, WA, USA; ^4^ Department of Psychiatry, Taipei Veterans General Hospital, Taipei, Taiwan; ^5^ Division of Psychiatry, National Yang-Ming University, Taipei, Taiwan; ^6^ Department of Public Health, Institute of Epidemiology and Preventive Medicine, National Taiwan University, Taipei, Taiwan; ^7^ Center for Neuropsychiatric Research, National Health Research Institutes, Miaoli County, Taiwan; ^8^ Department of Agronomy, College of Agriculture & Natural Resources, National Chung Hsing University, Taichung, Taiwan

**Keywords:** Alzheimers diseases, cognitive aging, gene-gene and gene-lifestyle interactions, Mini-Mental State Examination, single nucleotide polymorphisms, Gerotarget

## Abstract

Genome-wide association studies and meta-analyses implicated that increased risk of developing Alzheimers diseases (AD) has been associated with the *ABCA7*, *APOE*, *BIN1*, *CASS4*, *CD2AP*, *CD33*, *CELF1*, *CLU*, *CR1*, *DSG2*, *EPHA1*, *FERMT2*, *HLA-DRB1*, *HLA-DRB4*, *INPP5D*, *MEF2C*, *MS4A4A*, *MS4A4E*, *MS4A6E*, *NME8*, *PICALM*, *PLD3*, *PTK2B*, *RIN3*, *SLC24A4*, *SORL1*, and *ZCWPW1* genes. In this study, we assessed whether single nucleotide polymorphisms (SNPs) within these 27 AD-associatedgenes are linked with cognitive aging independently and/or through complex interactions in an older Taiwanese population. We also analyzed the interactions between lifestyle and these genes in influencing cognitive aging. A total of 634 Taiwanese subjects aged over 60 years from the Taiwan Biobank were analyzed. Mini-Mental State Examination (MMSE) scores were performed for all subjects to evaluate cognitive functions. Out of the 588 SNPs tested in this study, only the association between *CASS4*-rs911159 and cognitive aging persisted significantly (*P* = 2.2 × 10^−5^) after Bonferroni correction. Our data also showed a nominal association of cognitive aging with the SNPs in six more key AD-associated genes, including *EPHA1-*rs10952552, *FERMT2-*rs4901317, *MEF2C-*rs9293506, *PLD3-*rs11672825, *RIN3-*rs1885747, and *SLC24A4*-rs67063100 (*P* = 0.0018∼0.0097). Additionally, we found the interactions among *CASS4-*rs911159, *EPHA-*rs10952552, *FERMT2-*rs4901317, *MEF2C-*rs9293506, or *SLC24A4-*rs67063100 on cognitive aging (*P* = 0.004∼0.035). Moreover, our analysis suggested the interactions of *SLC24A4-*rs67063100 or *MEF2C*-rs9293506 with lifestyle such as alcohol consumption, smoking status, physical activity, or social support on cognitive aging (*P* = 0.008∼0.041). Our study indicates that the AD-associated genes may contribute to the risk of cognitive aging independently as well as through gene-gene and gene-lifestyle interactions.

## INTRODUCTION

Cognitive aging is considered as a gradual and ongoing process of change in cognitive capacity with advancing age [1]. It is worth mentioning that cognitive aging may increase the likelihood of several neurodegenerative disorders, such as mild cognitive impairment, dementia, and Alzheimer's diseases (AD) since prior work has estimated that rates of neurodegenerative disorders increase exponentially with age [2]. With ever-increasing elder populations not only in affluent societies but also in developing countries, the pervasiveness of neurodegenerative disorders has turned out to be a mammoth public health issue [3]. In this regard, biomarkers have become increasingly essential to grasp the biology of cognitive aging [4]. The search for biomarkers for cognitive aging has been active, and the same biomarkers for AD are also commonly employed in cognitive aging research due to a high prevalence of AD in the older adults [5].

Several genome-wide association studies (GWAS) indicated that single nucleotide polymorphisms (SNPs) within 11 genes are associated with AD risk, including the ATP binding cassette subfamily A member 7 (*ABCA7*), apolipoprotein E (*APOE*), bridging integrator 1 (*BIN1*), CD2 associated protein (*CD2AP*), CD33 molecule (*CD33*), clusterin (*CLU*), complement C3b/C4b receptor 1 (*CR1*), EPH receptor A1 (*EPHA1*), membrane spanning 4-domains A4A (*MS4A4A*), membrane spanning 4-domains A4E (*MS4A4E*), membrane spanning 4-domains A6E (*MS4A6E*), and phosphatidylinositol binding clathrin assembly protein (*PICALM*) genes [6–10]. The subsequent meta-analysis of GWAS studies (*n* = 74,046) by Lambert et al. further tracked down 14 AD risk genes, including the Cas scaffolding protein family member 4 (*CASS4*), CUGBP Elav-like family member 1 (*CELF1*), desmoglein 2 (*DSG2*), fermitin family member 2 (*FERMT2*), major histocompatibility complex class II DR beta 1 (*HLA-DRB1*), major histocompatibility complex class II DR beta 4 (*HLA-DRB4*), inositol polyphosphate-5-phosphatase D (*INPP5D*), myocyte enhancer factor 2C (*MEF2C*), NME/NM23 family member 8 (*NME8*), protein tyrosine kinase 2 beta (*PTK2B*), Ras and Rab interactor 3 (*RIN3*), solute carrier family 24 member 4 (*SLC24A4*), sortilin related receptor 1 (*SORL1*), zinc finger CW-type and PWWP domain containing 1 (*ZCWPW1*) [11]. Additionally, Cruchaga et al. performed whole-exome sequencing and identified the phospholipase D family member 3 (*PLD3*) gene as an AD risk gene [12]. Recent epistasis studies further indicated that the *CLU*-*MS4A4E* [13, 14] and *CD33*-*MS4A4E* [13] gene-gene interactions may have a major impact in modulating AD susceptibility. By using the established AD-associated genes, Chibnik et al. also found an association of cognitive decline with the *CR1* rs6656401 SNP, but not with the *CLU* rs11136000 and *PICALM* rs7110631 SNPs [15]. Moreover, Nettiksimmons et al. utilized the AD-associated genes and demonstrated that the *ABCA7* rs3764650 and *CD33* rs3865444 SNPs were associated with cognitive decline in the female cohort of Caucasian older adults, but not in the male cohort [16].

While several encouraging findings on the relationship between the AD-associated genes and cognitive aging have emerged, to our knowledge, human data is scarce in terms of single nucleotide polymorphisms (SNPs). Moreover, lifestyle factors such as alcohol consumption, smoking status, physical activity, and social support have not received as much attention as genetic factors in cognitive aging research, and thus the interplay between the AD-associated genes and lifestyle should be thoroughly investigated. Given that gene-gene and gene-lifestyle interactions may play a key role in the development of cognitive aging, we hypothesized that the AD-associated genes may contribute to the etiology of cognitive aging independently and/or through complex interactions. The gene panel encompasses 27 aforementioned AD-associated genes (Supplementary Table 1), including the *ABCA7*, *APOE*, *BIN1*, *CASS4*, *CD2AP*, *CD33*, *CELF1*, *CLU*, *CR1*, *DSG2*, *EPHA1*, *FERMT2*, *HLA-DRB1*, *HLA-DRB4*, *INPP5D*, *MEF2C*, *MS4A4A*, *MS4A4E*, *MS4A6E*, *NME8*, *PICALM*, *PLD3*, *PTK2B*, *RIN3*, *SLC24A4*, *SORL1*, and *ZCWPW1* genes.

## RESULTS

Table 1 describes the demographic and clinical characteristics of the study population, including 634 subjects. The median MMSE score was 27 and interquartile range was 25-29.

**Table 1:** Demographic and clinical characteristics of study subjects

Characteristic	Overall
No. of subjects, n	634
Mean age ± SD, years	64.2±2.9
Male, %/Female, %	50.16/49.84
Married, %	82.01
Living alone, %	8.51
Any physical activity, %	63.72
Current alcohol drinker, %	5.52
Current smoker, %	6.46
High school graduate, %	59.30
MMSE score, median (IQR)	27 (25–29)

IQR = interquartile range, MMSE = Mini-Mental State Examination, SD = standard deviation.

Data are presented as mean ± standard deviation.

First, we investigated the association between cognitive aging and 27 AD-associated genes. Among the 588 SNPs assessed in this study (Supplementary Table S1), there were 63 SNPs in the 17 AD-associated genes showing an evidence of association (*P* < 0.05) with MMSE scores as shown in Table 2. However, only the association of the *CASS4* rs911159 SNP with MMSE scores reached a significance after Bonferroni correction, where the three separate genetic models were taken into account (*P* < 0.05/(586 × 3) = 2.8 × 10^−5^). As demonstrated in Table 2, the *CASS4* rs911159 SNP indicated an association with MMSE scores among subjects after adjustment of covariates such as age, gender, and education for genetic models, including the additive model (*P* = 2.2 × 10^−5^) and recessive model (*P* = 2.2 × 10^−5^).

**Table 2:** Linear regression models of associations between the MMSE scores and 17 selective AD-related genes, which have an evidence of association (*P* < 0.05)

						Additive model			Recessive model			Dominant model		
Gene	CHR	SNP	A1	A2	MAF	BETA	SE	*P*	BETA	SE	*P*	BETA	SE	*P*
*CASS4*	20	rs11698292	C	T	0.13	-1.50	0.53	**0.0051**	-3.00	1.06	**0.0051**	-0.16	0.26	0.5446
		rs17365060	G	A	0.15	-1.09	0.43	0.0114	-2.17	0.86	0.0118	-0.19	0.25	0.4350
		rs6069746	C	T	0.16	-1.21	0.43	**0.0051**	-2.37	0.86	**0.0060**	-0.33	0.25	0.1917
		rs911159	A	G	0.15	-2.13	0.50	**2.2 × 10**^−5^	-4.24	0.99	**2.2 × 10**^−5^	-0.25	0.25	0.3264
*CD2AP*	6	rs1485785	C	T	0.40	-0.16	0.17	0.3285	0.03	0.30	0.9296	-0.53	0.24	0.0259
		rs28360587	G	T	0.39	-0.16	0.17	0.3361	0.04	0.30	0.8859	-0.55	0.24	0.0212
		rs9357542	G	A	0.30	-0.27	0.20	0.1837	-0.28	0.38	0.4709	-0.53	0.22	0.0182
*CD33*	19	rs1354106	G	T	0.23	0.49	0.30	0.1041	0.81	0.60	0.1791	0.54	0.23	0.0198
		rs1803254	C	G	0.31	0.21	0.21	0.3331	0.17	0.41	0.6759	0.50	0.22	0.0258
*CR1*	1	rs12033963	A	G	0.23	0.44	0.27	0.0998	0.73	0.53	0.1654	0.47	0.23	0.0430
		rs12034383	A	G	0.37	-0.19	0.18	0.2753	-0.13	0.33	0.6941	-0.48	0.23	0.0357
*EPHA1*	7	rs10952552	A	G	0.22	0.72	0.24	**0.0026**	1.47	0.47	**0.0018**	0.15	0.23	0.5023
*FERMT2*	14	rs4901317	C	T	0.05	-2.34	0.81	**0.0041**	-4.62	1.63	**0.0047**	-0.85	0.35	0.0155
*MEF2C*	5	rs11949307	T	G	0.18	-0.74	0.37	0.0466	-1.54	0.74	0.0374	0.07	0.24	0.7665
		rs770463	T	C	0.35	-0.32	0.18	0.0706	-0.77	0.33	0.0206	0.05	0.23	0.8429
		rs7737567	T	C	0.12	-1.22	0.58	0.0360	-2.39	1.16	0.0393	-0.27	0.26	0.2992
		rs9293506	T	C	0.12	-1.43	0.54	**0.0081**	-2.80	1.07	**0.0092**	-0.33	0.26	0.2157
*MS4A4A*	11	rs12283601	A	G	0.28	-0.42	0.21	0.0401	-0.70	0.40	0.0794	-0.44	0.22	0.0495
		rs1365246	C	T	0.17	-0.23	0.35	0.5189	-0.30	0.70	0.6639	-0.53	0.25	0.0368
		rs7104122	C	G	0.17	-0.28	0.33	0.3938	-0.43	0.66	0.5143	-0.51	0.25	0.0402
*MS4A4E*	11	rs4939320	C	T	0.31	0.35	0.19	0.0612	0.52	0.36	0.1484	0.47	0.23	0.0419
		rs607639	A	G	0.16	-0.79	0.37	0.0318	-1.47	0.74	0.0461	-0.56	0.25	0.0263
		rs650853	T	C	0.16	-0.71	0.36	0.0464	-1.32	0.71	0.0657	-0.55	0.25	0.0309
		rs662674	A	G	0.16	-0.74	0.38	0.0544	-1.37	0.76	0.0734	-0.51	0.25	0.0439
		rs718376	A	G	0.31	0.35	0.18	0.0562	0.49	0.35	0.1567	0.50	0.22	0.0268
*MS4A6E*	11	rs11230281	C	A	0.49	-0.19	0.16	0.2208	-0.52	0.26	0.0448	0.01	0.26	0.9815
		rs2289612	A	C	0.38	-0.33	0.17	0.0571	-0.38	0.32	0.2365	-0.57	0.23	0.0128
		rs2289614	C	G	0.10	0.44	0.47	0.3481	0.80	0.95	0.3989	0.61	0.29	0.0399
*PICALM*	11	rs11234495	T	C	0.50	-0.25	0.16	0.1150	-0.13	0.26	0.6105	-0.54	0.26	0.0391
		rs592314	A	G	0.33	0.40	0.20	0.0426	0.89	0.38	0.0187	0.00	0.22	0.9974
		rs669556	C	T	0.33	0.40	0.20	0.0473	0.86	0.38	0.0261	0.04	0.23	0.8591
*PLD3*	19	rs11672825	T	G	0.50	0.38	0.16	0.0160	0.34	0.26	0.1928	0.68	0.25	**0.0071**
		rs4490097	A	C	0.47	0.28	0.16	0.0828	0.27	0.27	0.3329	0.49	0.25	0.0488
		rs7507651	T	G	0.47	0.30	0.16	0.0600	0.27	0.27	0.3177	0.54	0.25	0.0278
*PTK2B*	8	rs10109834	C	A	0.16	0.75	0.36	0.0372	1.51	0.71	0.0346	0.09	0.25	0.7298
*RIN3*	14	rs10467865	G	A	0.22	0.31	0.26	0.2216	0.46	0.50	0.3673	0.50	0.23	0.0294
		rs11627032	C	T	0.28	-0.34	0.22	0.1285	-0.49	0.44	0.2603	-0.47	0.22	0.0349
		rs12884739	A	G	0.41	0.19	0.16	0.2369	0.57	0.29	0.0500	-0.14	0.23	0.5464
		rs1885747	G	A	0.18	-0.90	0.35	**0.0097**	-1.69	0.69	0.0150	-0.49	0.24	0.0399
		rs4904957	A	G	0.38	-0.20	0.18	0.2496	-0.05	0.33	0.8781	-0.59	0.23	0.0103
		rs4904960	C	T	0.38	0.38	0.18	0.0329	0.69	0.32	0.0346	0.26	0.23	0.2626
		rs8012413	A	G	0.14	-0.01	0.35	0.9853	0.15	0.70	0.8283	-0.56	0.25	0.0245
		rs8017311	A	G	0.35	0.43	0.18	0.0166	0.72	0.34	0.0320	0.40	0.23	0.0771
		rs9323880	T	C	0.22	0.42	0.26	0.1081	0.70	0.51	0.1738	0.47	0.23	0.0434
		rs943655	A	C	0.32	0.42	0.19	0.0313	0.82	0.37	0.0270	0.19	0.23	0.4052
		rs9788457	C	T	0.26	-0.22	0.23	0.3197	-0.23	0.44	0.6101	-0.53	0.22	0.0189
*SLC24A4*	14	rs72631607	A	G	0.48	0.22	0.16	0.1548	0.56	0.26	0.0321	0.03	0.25	0.9177
		rs8022236	A	G	0.31	-0.31	0.22	0.1643	-0.86	0.43	0.0436	0.35	0.23	0.1360
		rs12435024	A	G	0.32	-0.51	0.19	**0.0065**	-0.99	0.35	**0.0054**	-0.26	0.23	0.2517
		rs10138927	C	G	0.20	-0.58	0.28	0.0378	-1.14	0.56	0.0411	-0.24	0.24	0.3164
		rs10431740	T	C	0.24	-0.51	0.18	**0.0055**	-1.03	0.36	**0.0041**	-0.28	0.23	0.2292
		rs61977311	A	G	0.23	-0.71	0.27	**0.0099**	-1.22	0.54	0.0256	-0.64	0.23	**0.0049**
		rs67063100	A	G	0.17	-0.66	0.33	0.0447	-1.15	0.66	0.0811	-0.71	0.24	**0.0038**
		rs11160069	T	C	0.45	-0.35	0.17	0.0380	-0.47	0.30	0.1208	-0.46	0.24	0.0537
		rs12434016	G	T	0.38	-0.49	0.17	**0.0041**	-0.73	0.31	0.0194	-0.58	0.23	0.0127
		rs10431637	C	A	0.15	-0.63	0.38	0.0998	-1.13	0.76	0.1395	-0.58	0.25	0.0199
		rs78739077	T	G	0.11	-1.42	0.63	0.0253	-2.77	1.26	0.0287	-0.42	0.27	0.1273
*SORL1*	11	rs3781825	C	T	0.13	0.25	0.45	0.5769	0.67	0.90	0.4597	-0.63	0.26	0.0163
		rs3862605	C	T	0.17	0.33	0.29	0.2521	0.55	0.58	0.3450	0.49	0.25	0.0460
		rs4631890	A	G	0.30	0.30	0.21	0.1456	0.81	0.40	0.0441	-0.25	0.22	0.2666
		rs4936632	G	A	0.43	-0.09	0.16	0.6016	0.25	0.29	0.3854	-0.53	0.25	0.0330
		rs661057	T	C	0.45	-0.02	0.16	0.8823	0.39	0.27	0.1551	-0.51	0.24	0.0373
*ZCWPW1*	7	rs5015755	T	G	0.15	0.74	0.35	0.0352	1.49	0.69	0.0321	0.08	0.25	0.7478

A1 = minor allele, A2 = major allele, AD = Alzheimer's disease, BETA = Beta coefficients, Chr = chromosome, MAF = minor allele frequency, MMSE = Mini-Mental State Examination, SE = standard error.

Analysis was obtained after adjustment for covariates including age, gender, and education. *P* values of < 0.01 are shown in bold.

Furthermore, the distribution of *APOE* alleles (ε2, ε3, and ε4) in our sample was 6.2%, 82.7%, and 11.1%, respectively (Supplementary Table S2A). Additionally, we found that the presence of the *APOE* ε4 allele had no significant effects in MMSE scores (Supplementary Table S2B). We also examined whether the genetic load of *APOE* ε4 (that is, non-carrier, heterozygous, and homozygous for the ε4 allele) has a significant impact in MMSE scores. Our results revealed that MMSE scores were associated with neither *APOE* ε4 homozygotes nor heterozygotes (Supplementary Table S2B).

Then, we identified a nominal association of MMSE scores with 12 more SNPs, including *CASS4* (rs11698292, rs6069746), *EPHA1* rs10952552, *FERMT2* rs4901317, *MEF2C* rs9293506, *PLD3* rs11672825, *RIN3* rs1885747, and *SLC24A4* (rs12435024, rs10431740, rs61977311, rs67063100, rs12434016) (Table 2). For further investigation in the subsequent analyses, we selected seven key SNPs in seven AD-associated genes with evidence of association, including *CASS4* rs911159 (*P* = 2.2 × 10^−5^), *EPHA1* rs10952552 (*P* = 0.0018), *FERMT2* rs4901317 (*P* = 0.0041), *MEF2C* rs9293506 (*P* = 0.0081), *PLD3* rs11672825 (*P* = 0.0071), *RIN3* rs1885747 (*P* = 0.0097), and *SLC24A4* rs67063100 (*P* = 0.0038). In addition, the genotype frequency distributions for the *CASS4* rs911159, *EPHA1* rs10952552, *FERMT2* rs4901317, *MEF2C* rs9293506, *PLD3* rs11672825, *RIN3* rs1885747, and *SLC24A4* rs67063100 SNPs were in accordance with the Hardy-Weinberg equilibrium among the subjects (*P* = 0.126, 0.253, 0.611, 0.271, 0.800, 0.416, and 0.649, respectively).

Next, we employed categorized MMSE scores as an outcome (normal: MMSE score ≥ 24; cognitive impairment: MMSE score < 24) for gene-gene and gene-lifestyle analysis. First, the GMDR analysis was used to assess the impacts of combinations between the seven key SNPs in cognitive aging including age, gender, and education as covariates. Table 3 summarizes the results obtained from GMDR analysis for two-way gene-gene interaction models with covariate adjustment. As shown in Table 3, there was a significant two-way model involving *CASS4* rs911159 and *SLC24A4* rs67063100 (*P* = 0.035), *EPHA1* rs10952552 and *SLC24A4* rs67063100 (*P* = 0.016), *FERMT2* rs4901317 and *MEF2C* rs9293506 (*P* = 0.008), *FERMT2* rs4901317 and *SLC24A4* rs67063100 (*P* = 0.004), as well as *MEF2C* rs9293506 and *SLC24A4* rs67063100 (*P* = 0.009), indicating a potential gene-gene interaction between *CASS4* and *SLC24A4*, between *EPHA1* and *SLC24A4*, between *FERMT2* and *MEF2C*, between *FERMT2* and *SLC24A4*, as well as between *MEF2C* and *SLC24A4* in influencing cognitive aging.

**Table 3:** Gene-gene interaction models identified by the GMDR method with adjustment for age, gender, and education

Interaction model	Testing accuracy (%)	*P* value
*CASS4* rs911159, *EPHA1* rs10952552	52.56	0.259
*CASS4* rs911159, *FERMT2* rs4901317	53.57	0.139
*CASS4* rs911159, *MEF2C* rs9293506	52.52	0.262
*CASS4* rs911159, *PLD3* rs11672825	55.85	0.059
*CASS4* rs911159, *RIN3* rs1885747	48.31	0.699
*CASS4* rs911159, *SLC24A4* rs67063100	56.53	**0.035**
*EPHA1* rs10952552, *FERMT2* rs4901317	46.39	0.840
*EPHA1* rs10952552, *MEF2C* rs9293506	55.12	0.093
*EPHA1* rs10952552, *PLD3* rs11672825	51.36	0.361
*EPHA1* rs10952552, *RIN3* rs1885747	49.35	0.626
*EPHA1* rs10952552, *SLC24A4* rs67063100	57.69	**0.016**
*FERMT2* rs4901317, *MEF2C* rs9293506	57.47	**0.008**
*FERMT2* rs4901317, *PLD3* rs11672825	49.84	0.519
*FERMT2* rs4901317, *RIN3* rs1885747	48.37	0.691
*FERMT2* rs4901317, *SLC24A4* rs67063100	58.48	**0.004**
*MEF2C* rs9293506, *PLD3* rs11672825	55.02	0.104
*MEF2C* rs9293506, *RIN3* rs1885747	49.65	0.570
*MEF2C* rs9293506, *SLC24A4* rs67063100	58.05	**0.009**
*PLD3* rs11672825, *RIN3* rs1885747	48.96	0.600
*PLD3* rs11672825, *SLC24A4* rs67063100	54.65	0.142
*RIN3* rs1885747, *SLC24A4* rs67063100	55.34	0.085

GMDR = generalized multifactor dimensionality reduction.

*P* value was based on 1,000 permutations. Analysis was obtained after adjustment for covariates including age, gender, and education. *P* values of < 0.05 are shown in bold.

Furthermore, we utilized multivariable logistic regression analysis with adjustment for age, gender, and education to assess the two-way gene-gene interaction models selected by the GMDR method (Supplementary Table S3). Our analysis revealed that the carriers with the AA genotype of *CASS4* rs911159 and the GG genotype of *SLC24A4* rs67063100 had a 7.05-fold increased risk for cognitive aging, compared to those with the GG genotype of *CASS4* rs911159 and the GG genotype of *SLC24A4* rs67063100 (Supplementary Table S3). Additionally, the carriers with the AG genotype of *EPHA1* rs10952552 and the A allele of *SLC24A4* rs67063100 had a 2.26-fold increased risk for cognitive aging, compared to those with the GG genotype of *EPHA1* rs10952552 and the GG genotype of *SLC24A4* rs67063100 (Supplementary Table S3). Moreover, the carriers with the TT genotype of *FERMT2* rs4901317 and the GG genotype of *SLC24A4* rs67063100 had a 0.23-fold increased risk for cognitive aging, compared to those with the CT genotype of *FERMT2* rs4901317 and the A allele of *SLC24A4* rs67063100 (Supplementary Table S3). Similarly, the carriers with the TC genotype of *MEF2C* rs9293506 and the A allele of *SLC24A4* rs67063100 had a 2.79-fold increased risk for cognitive aging, compared to those with the CC genotype of *MEF2C* rs9293506 and the GG genotype of *SLC24A4* rs67063100 (Supplementary Table S3).

Furthermore, statistical power analysis revealed that the present study had a 99.9% power to detect gene-gene interactions between *CASS4* and *SLC24A4*, between *EPHA1* and *SLC24A4*, between *FERMT2* and *SLC24A4*, as well as between *MEF2C* and *SLC24A4*. In addition, this study had a 91.3% power to detect a gene-gene interaction between *FERMT2* and *MEF2C*.

Moreover, Table 4 shows the GMDR analysis of gene-lifestyle interaction models in cognitive aging using age, gender, and education as covariates. As shown in Table 4, there was a significant two-way model involving *SLC24A4* rs67063100 and lifestyle factors such as smoking (*P* = 0.041), alcohol consumption (*P* = 0.008), and physical activity (*P* = 0.038), indicating a potential gene-lifestyle interaction among *SLC24A4* and lifestyle factors in influencing cognitive aging. Similarly, there was a significant two-way model involving *MEF2C* rs9293506 and social support (*P* = 0.039). However, there was no significant two-way model involving lifestyle factors and other five SNPs including *CASS4* rs911159, *EPHA1* rs10952552, *FERMT2* rs4901317, *PLD3* rs11672825, and *RIN3* rs1885747.

**Table 4:** Gene-lifestyle interaction models identified by the GMDR method with adjustment for age, gender, and education

Interaction model	Testing accuracy (%)	*P* value
*CASS4* rs911159, smoking	50.85	0.416
*CASS4* rs911159, alcohol consumption	52.15	0.287
*CASS4* rs911159, physical activity	50.03	0.520
*CASS4* rs911159, social support	52.68	0.233
*EPHA1* rs10952552, smoking	52.40	0.275
*EPHA1* rs10952552, alcohol consumption	52.74	0.247
*EPHA1* rs10952552, physical activity	47.16	0.766
*EPHA1* rs10952552, social support	53.76	0.156
*FERMT2* rs4901317, smoking	50.75	0.388
*FERMT2* rs4901317, alcohol consumption	50.25	0.461
*FERMT2* rs4901317, physical activity	45.70	0.862
*FERMT2* rs4901317, social support	50.99	0.388
*MEF2C* rs9293506, smoking	54.39	0.081
*MEF2C* rs9293506, alcohol consumption	53.75	0.147
*MEF2C* rs9293506, physical activity	52.53	0.282
*MEF2C* rs9293506, social support	55.65	**0.039**
*PLD3* rs11672825, smoking	47.86	0.690
*PLD3* rs11672825, alcohol consumption	50.39	0.449
*PLD3* rs11672825, physical activity	49.04	0.598
*PLD3* rs11672825, social support	49.87	0.535
*RIN3* rs1885747, smoking	45.49	0.899
*RIN3* rs1885747, alcohol consumption	46.39	0.838
*RIN3* rs1885747, physical activity	46.13	0.857
*RIN3* rs1885747, social support	47.95	0.723
*SLC24A4* rs67063100, smoking	55.86	**0.041**
*SLC24A4* rs67063100, alcohol consumption	57.64	**0.008**
*SLC24A4* rs67063100, physical activity	56.81	**0.038**
*SLC24A4* rs67063100, social support	54.25	0.126

GMDR = generalized multifactor dimensionality reduction.

*P* value was based on 1,000 permutations. Analysis was obtained after adjustment for covariates including age, gender, and education. *P* values of < 0.05 are shown in bold.

## DISCUSSION

Our study is the first to date to pinpoint whether the main effects of 588 SNPs in 27 AD-associated genes are significantly associated with the risk of cognitive aging independently and/or through gene-gene interactions among old Taiwanese individuals. We also looked over the relationship between these genes and lifestyle factors to investigate whether these genes confer a risk of cognitive aging according to its impact on gene-lifestyle interactions. Here, we report for the first time that several SNPs of the AD-associated genes including *CASS4* rs911159, *EPHA1* rs10952552, *FERMT2* rs4901317, *MEF2C* rs9293506, *PLD3* rs11672825, *RIN3* rs1885747, and *SLC24A4* rs67063100 may play an important role in the modulation of cognitive aging in old adults in a Taiwanese population. Notably, the significant association of the *CASS4* rs911159 SNP with MMSE scores persisted after correction for multiple testing (*P* < 2.8 × 10^−5^). Additionally, our data revealed that gene-gene interactions of *EPHA1*, *MEF2C*, and *SLC24A4* may contribute to the etiology of cognitive aging. Our data also indicated that there were gene-lifestyle interactions of *SLC24A4* with lifestyle, such as smoking status, alcohol consumption, or physical activity. Finally, there was a gene-lifestyle interaction of *MEF2C* with social support.

To our knowledge, our results are the first to raise the possibility that 4 SNPs in the *CASS4* gene may contribute to the susceptibility for cognitive aging. Intriguingly, the *CASS4* rs911159 SNP (*P* = 2.2 × 10^−5^) persisted a significant association with MMSE scores after Bonferroni correction. The *CASS4* gene is located on chromosome 20q13.31 and encodes a member of the Crk-associated substrate scaffolding protein family [17]. The protein encoded by the *CASS4* gene has been implicated in the regulation of cell spreading, focal adhesion integrity, and focal adhesion kinase activation [17]. Furthermore, we speculate that the *CASS4* gene may play a central role in the amyloid precursor protein (APP) and Tau protein, which are the hallmarks of AD [18]. The meta-analysis of GWAS studies by Lambert et al. identified the *CASS4* rs7274581 SNP as an AD risk variant [11]. In the following GWAS study, Beecham et al. confirmed an association between *CASS4* rs7274581 and AD by using brain autopsy data [19]. On the contrary, Ruiz et al. suggested that the *CASS4* rs7274581 polymorphism was unlikely to influence AD in a Spanish sample in the following replication study [20]. Furthermore, Rosenthal et al. reported a major involvement of the *CASS4* rs6024870 polymorphism in AD in another replication study by using the RegulomeDB database [21]. Wang et al. also demonstrated an association between *CASS4* rs16979934 and AD in a USA sample in a subsequent GWAS study [22]. Finally, it should be noted that the minor allele frequencies of the imputed *CASS4* rs7274581, rs16979934, and rs6024870 SNPs are all 0% in this study (Supplementary Table S4).

The second locus, the rs10952552 SNP, was identified at the *EPHA1* gene. The *EPHA1* gene is located on chromosome 7q34-35 and encodes a member of the ephrins family of tyrosine kinase receptors, which have been indicated in mediating developmental events in the nervous system [18]. Two GWAS studies by Naj et al. [9] and Hollingworth et al. [10] indicated that the *EPHA1* rs11767557 SNP may contribute to the reduced susceptibility for AD. Moreover, the following meta-analysis of GWAS studies implicated that the *EPHA1* rs11771145 may be involved with reduced AD susceptibility [11]. On the contrary, we failed to capture an association between the *EPHA1* rs11771145 SNP and cognitive aging.

In addition, an intriguing finding was a positive association of cognitive aging with 11 SNPs within the *SLC24A4* gene, especially the rs12435024, rs10431740, rs61977311, rs67063100, and rs12434016 SNPs. The *SLC24A4* gene, located on chromosome 14q32.12, encodes a member of the potassium-dependent sodium/calcium exchanger protein family, which might be connected to neurological development [23]. In a meta-analysis study, Lambert et al. pinpointed *SLC24A4* rs10498633 as an AD risk SNP [11]; however, *SLC24A4* rs10498633 had no association with cognitive aging in our study.

The fourth locus, the rs4901317 SNP, was within the *FERMT2* gene, which is located on chromosome 14q22.1. The corresponding protein of the *FERMT2* gene has been previously implicated with roles in cell adhesion and Tau neurotoxicity [24]. The *FERMT2* rs17125944 SNP has been reported to associate with AD susceptibility in a meta-analysis study [11], but *FERMT2* rs17125944 had no association with cognitive aging in our study.

On another note, there was an association of cognitive aging with 3 SNPs within the *PLD3* gene, particularly the rs11672825 SNP. The *PLD3* gene, located on chromosome 19q13.2, encodes a member of the phospholipase D family of enzymes, which influence processing of amyloid-beta precursor protein [12]. Cruchaga et al. identified a rare *PLD3* rs145999145 (Val232Met) as an AD risk variant in a whole-exome sequencing study [12]; however, the minor allele frequency of the imputed *PLD3* rs145999145 SNP is 0% in this study (Supplementary Table S4).

We also observed an association of cognitive aging with 4 SNPs within the *MEF2C* gene, notably the rs9293506 SNP. The *MEF2C* gene, located on chromosome 5q14.3, encodes a member of the MADS box transcription enhancer factor 2 family of proteins, which plays a major role in hippocampal synaptic connectivity and thus may regulate hippocampal-dependent learning and memory [25]. The *MEF2C* rs190982 SNP has been demonstrated to link with AD in a meta-analysis study [11]. In contrast, *MEF2C* rs190982 showed no association with cognitive aging in our study.

Furthermore, our analysis indicated a positive association of cognitive aging with 11 SNPs within the *RIN3* gene, especially the rs1885747 SNP. The *RIN3* gene, located on chromosome 14q32.12, is in the vicinity of the *SLC24A4* gene and encodes a member of the RIN family of Ras interaction-interference proteins, which interacts with the BIN1 protein that might be linked with an AD-relevant pathological process involving APP and Tau pathology [26].

Remarkably, another intriguing finding was that we further inferred the epistatic effects between *CASS4*, *EPHA1*, *FERMT2*, *MEF2C*, or *SLC24A4* in influencing cognitive aging by using the GMDR approach. To our knowledge, no other study has been conducted to weigh gene-gene interactions between these genes. Besides the statistical significance, the potential biological mechanism under the interaction models was our concern. The functional relevance of the interactive impact of *CASS4*, *EPHA1*, *FERMT2*, *MEF2C*, or *SLC24A4* on cognitive aging remains to be elucidated. We further speculate that the *CASS4*, *EPHA1*, *FERMT2*, *MEF2C*, or *SLC24A4* genes may be involved in the same pathways or pathology. Yu et al. found that DNA methylation in the *SLC24A4* gene was associated with pathological AD diagnosis, suggesting that altered methylation in the *SLC24A4* gene might involve Tau pathology [27]. In addition, the *SLC24A4* gene is located next to the *RIN3* gene, which interacts with the *BIN1* gene in the Tau, APP [26], and endocytosis [28] pathways. By putting together the previous findings, the *SLC24A4*, *FERMT2*, and *CASS4* genes have been implicated in Tau pathology [11, 26]. Similarly, the *SLC24A4* and *EPHA1* genes are involved in the pathway of endocytosis [18, 28]. The *EPHA1* and *MEF2C* genes are also implicated in the process of immune response and neuroinflammation [11, 18]. Furthermore, the *SLC24A4* and *CASS4* genes have been linked with the metabolism of APP [11, 26].

In the GMDR analysis of gene-lifestyle interactions, we tracked down the interplay between the *SLC24A4* gene and lifestyle such as smoking, alcohol consumption, and physical activity as well as the interplay between the *MEF2C* gene and social support. It has been pointed out that common diseases are known to have a major genetic contribution, but only a small proportion of complex diseases overall is explained by the established candidate genes, suggesting that the impact of lifestyle and gene-lifestyle interactions will be essential in future studies [13, 29].

It is worth mentioning that the well-known MMSE, the most widely used screening test of cognition, can be easily administered in about 5 to10 minutes; however, it has floor and ceiling effects, reducing variability in the data [30]. On the other hand, a well-validated scale in cognitive performance is the Alzheimer's Disease Assessment Scale - Cognitive section (ADAS-Cog), where a four-point change on ADAS-Cog has been established as a clinically important change in cognition [31]. But, ADAS-Cog takes around 40 minutes to administer, and its length makes ADAS-Cog unsuitable for clinical practice [30].

This study has both strengths and limitations. The main weakness was that our observations require much further research to pinpoint whether the present research findings are sustained in diverse ethnic groups [32–34]. Second, given that the mean age (±SD) of the sample was 64.2 (±2.9), our findings are not generalizable to much older cohorts that would be at the highest risk of developing neurodegenerative disorders. The outlook for prospective clinical trials with other ethnic populations is still warranted to provide a comprehensive evaluation of the association and interactions of the investigated variants with cognitive aging [35–37]. On the other hand, a key strength of our study was that we leveraged lifestyle data, which served a suitable opportunity to facilitate the interplay between the investigated variants and lifestyle factors.

## CONCLUSIONS

In conclusion, we explored an extensive analysis of the association as well as gene-gene and gene-lifestyle interactions of the AD-associated genes with cognitive aging in older Taiwanese subjects. Overall, results from the current study serve to highlight that the *CASS4*, *EPHA1*, *FERMT2*, *MEF2C*, *PLD3*, *RIN3*, and *SLC24A4* genes may affect the prevalence of cognitive aging independently and/or through complex gene-gene and gene-lifestyle interactions. Independent replication studies with a much larger number of participants will likely demonstrate further insights into the role of the cognitive aging-related genes tracked down in this study.

## MATERIALS AND METHODS

### Study population

This study incorporated Taiwanese Han Chinese subjects from the Taiwan Biobank [38–40]. The study cohort consisted of 634 participants. Ethical approval for the study was granted by the Internal Review Board of the Taiwan Biobank before conducting the study. Each subject signed the approved informed consent form. All experiments were performed in accordance with relevant guidelines and regulations.

Education was defined based on whether or not high school was attended. Current alcohol drinker was defined as currently drinking 150 ml of alcohol per week for more than six months. Current smoker was defined as currently smoking for more than six months. Physical activity was defined by the amount of exercise activity for more than three times and more than 30 minutes each time in each week. Social support was assessed based on marital status and whether or not living alone.

### Cognitive assessment

Global cognitive assessment was performed using the 30-point Mini-Mental State Examination (MMSE), which includes questions based on five domains such as orientation, registration, attention and calculation, recall, and language. We analyzed MMSE as a continuous outcome, as well as according to categories based on previously defined MMSE thresholds [30]: MMSE score ≥ 24 (normal) and MMSE score < 24 (cognitive impairment).

### Genotyping

DNA was isolated from blood samples using a QIAamp DNA blood kit following the manufacturer's instructions (Qiagen, Valencia, CA, USA). The quality of the isolated genomic DNA was evaluated using agarose gel electrophoresis, and the quantity was determined by spectrophotometry [41, 42]. SNP genotyping was carried out using the custom Taiwan BioBank chips and run on the Axiom Genome-Wide Array Plate System (Affymetrix, Santa Clara, CA, USA). The SNP panel covered variants from the following 27 AD-associated genes: *ABCA7*, *APOE*, *BIN1*, *CASS4*, *CD2AP*, *CD33*, *CELF1*, *CLU*, *CR1*, *DSG2*, *EPHA1*, *FERMT2*, *HLA-DRB1*, *HLA-DRB4*, *INPP5D*, *MEF2C*, *MS4A4A*, *MS4A4E*, *MS4A6E*, *NME8*, *PICALM*, *PLD3*, *PTK2B*, *RIN3*, *SLC24A4*, *SORL1*, and *ZCWPW1*.

In addition, *APOE* variants (ε2, ε3, and ε4) were derived from rs7412 and rs4420638, where rs4420638, a proxy for *APOE* rs429358, was used to impute rs429358 [43].

Moreover, we leveraged MACH [44] to carry out genotype imputation with 20 iterations of the Markov sampler, 200 states, and 1000 genomes reference panel. MACH employs a Markov Chain algorithm to impute missing genotypes by using haplotypes as templates [44].

### Statistical analysis

In this study, we weighed the association of the investigated SNP with MMSE scores by a general linear model using age, gender, education as covariates [45, 46]. The genotype frequencies were assessed for Hardy-Weinberg equilibrium using a χ^2^ goodness-of-fit test with 1 degree of freedom (i.e. the number of genotypes minus the number of alleles). Multiple testing was adjusted by the Bonferroni correction. The criterion for significance was set at *P* < 0.05 for all tests. Data are presented as the mean ± standard deviation.

To investigate gene-gene and gene-lifestyle interactions, we leveraged the generalized multifactor dimensionality reduction (GMDR) method [47]. We tested two-way interactions using 10-fold cross-validation. The GMDR software provides some output parameters, including the testing accuracy and empirical *P* values, to assess each selected interaction. Furthermore, the testing accuracy is a measure of the degree to which the interaction accurately predicts case-control status with scores between 50% (implying that the model predicts no better than chance) and 100% (implying perfect prediction). Moreover, we provided age, gender, education as covariates for gene-gene and gene-lifestyle interaction models in our interaction analyses. Permutation testing obtains empirical P values of prediction accuracy as a benchmark based on 1,000 shuffles.

Based on the effect sizes in this study, the power to detect gene-gene interactions was evaluated by QUANTO software (http://biostats.usc.edu/Quanto.html).

## SUPPLEMENTARY MATERIALS FIGURES AND TABLES


