# Supplementary material for: Association and interaction effects of Alzheimer’s disease-associated genes and lifestyle on cognitive aging in older adults in a Taiwanese population
Source: Oncotarget. 2017 Feb 11;8(15):24077–87. doi: 10.18632/oncotarget.15269 (PMC5421828; doi:10.18632/oncotarget.15269)
Supplement: Supplementary file 1 [file oncotarget-08-24077-s001.pdf]

Supplementary Table S1. Linear regression models of associations between the MMSE scores and 586 SNPs in 27 AD-related genes.

Gene	CHR	SNP	A1	A2	P (additive)	P (recessive)	P (dominant)
ABCA7	19	rs10419707	C	T	0.5939	0.6273	0.4829
		rs3752228	T	C	0.7855	0.8177	0.2417
		rs3764645	G	A	0.8627	0.7883	0.9456
		rs3752232	G	A	0.7837	0.8175	0.2167
		rs3764650	G	T	0.1196	0.1069	0.5117
		rs4147912	A	C	0.0991	0.1369	0.0828
		rs3764651	G	A	0.3223	0.4160	0.3461
		rs3752246	G	C	0.2116	0.1570	0.7598
BIN1	2	rs880436	T	C	0.5159	0.6578	0.5182
		rs1060743	G	A	0.1765	0.3502	0.1911
		rs13430398	T	C	0.7979	0.8240	0.4081
		rs10166461	A	G	0.9106	0.9196	0.7997
CASS4	20	rs6127744	T	G	0.3067	0.4335	0.3106
		rs11698292	C	T	0.0051	0.0051	0.5446
		rs17365060	G	A	0.0114	0.0118	0.4350
		rs735780	G	A	0.8787	0.8344	0.5423
		rs911159	A	G	2.2×10^{-5}	2.2×10^{-5}	0.3264
		rs6024877	C	T	0.9169	0.9106	0.7484
		rs3787390	A	G	0.4958	0.8604	0.1924
		rs6069746	C	T	0.0051	0.0060	0.1917
		rs1884910	C	G	0.2339	0.2804	0.4124
		rs3746624	C	T	0.0784	0.1525	0.0746
CD2AP	6	rs9357542	G	A	0.1837	0.4709	0.0182
		rs9349409	T	G	0.5505	0.6375	0.0772
		rs200869952	T	C	0.4940	0.5728	0.0929
		rs9395279	T	C	0.5587	0.6425	0.0877
		rs9395284	A	G	0.4615	0.5166	0.2744
		rs72871200	T	G	0.5839	0.6199	0.2751
		rs1485785	C	T	0.3285	0.9296	0.0259

		rs28360587	G	T	0.3361	0.8859	0.0212
		rs10948368	C	T	0.4607	0.5165	0.2737
CD33	19	rs1399837	C	G	0.7453	0.7481	0.7746
		rs3826656	A	G	0.3420	0.5538	0.0549
		rs1710398	C	A	0.7422	0.7456	0.7389
		rs3865444	A	C	0.1251	0.1523	0.1397
		rs1354106	G	T	0.1041	0.1791	0.0198
		rs1399839	C	A	0.6767	0.6726	0.9442
		rs273653	A	G	0.6801	0.6726	0.8348
		rs1803254	C	G	0.3331	0.6759	0.0258
CELF1	11	rs7933019	C	G	0.2383	0.4082	0.1883
		rs11039260	A	C	0.8276	0.8932	0.6173
		rs61895112	C	T	0.5707	0.8441	0.1496
CLU	8	rs2279590	T	C	0.8584	0.9056	0.6415
		rs9331908	C	T	0.7750	0.8389	0.5396
		rs11136000	T	C	0.7819	0.8396	0.5735
		rs1532278	T	C	0.9360	0.9924	0.6315
		rs9331888	G	C	0.2356	0.7134	0.1069
		rs9314349	G	A	0.2817	0.2756	0.9820
CR1	1	rs7525160	C	G	0.2022	0.2820	0.3076
		rs112822892	G	A	0.7589	0.7266	0.5921
		rs112741151	A	G	0.8683	0.8349	0.5366
		rs113830567	T	C	0.5415	0.7082	0.3481
		rs11371768	A	N	0.5755	0.8285	0.1636
		rs61822967	A	G	0.7935	0.5183	0.2920
		rs2274566	T	C	0.7139	0.9082	0.1880
		rs12034598	G	A	0.3674	0.6231	0.0856
		rs646817	G	A	0.4066	0.7016	0.0722
		rs10779330	A	G	0.4372	0.7222	0.0866
		rs12141045	C	T	0.4464	0.5987	0.2934
		rs7539922	A	G	0.5384	0.7941	0.1822
		rs677066	C	T	0.5690	0.8481	0.1914
		rs11118167	C	T	0.3289	0.4047	0.3434

		rs6691117	G	A	0.5004	0.7652	0.1574
		rs3818361	A	G	0.8469	0.8254	0.3051
		rs7542544	A	C	0.7855	0.7515	0.1484
		rs6701713	A	G	0.8568	0.8105	0.3076
		rs117590864	A	C	NA	NA	0.4510
		rs1408078	T	C	0.9144	0.6406	0.4298
		rs17048010	C	T	0.8346	0.9339	0.3990
		rs12034383	A	G	0.2753	0.6941	0.0357
		rs12033963	A	G	0.0998	0.1654	0.0430
DSG2	18	rs9945031	A	G	0.5471	0.8797	0.2653
		rs9304099	T	G	0.5727	0.8690	0.3121
		rs78454147	T	C	0.1456	0.1359	0.4968
		rs11081701	C	T	0.8024	0.9270	0.6708
		rs56009511	A	G	0.8363	0.8075	0.6218
		rs2155948	T	G	0.8642	0.9885	0.7441
		rs2276149	A	G	0.6533	0.5890	0.9520
		rs9807379	G	A	0.8800	0.9548	0.4852
		rs2230233	T	C	0.9683	0.9112	0.9605
		rs62095194	A	G	0.9637	0.9053	0.8275
		rs10502572	T	C	0.8222	0.8122	0.9348
		rs10502573	A	G	0.5513	0.2429	0.7437
		rs2290128	T	C	0.5414	0.2411	0.7554
		rs1042769	G	A	0.4945	0.4884	0.9009
		rs1791235	T	C	0.4614	0.1804	0.1318
		rs11542765	G	A	0.7953	0.3950	0.6871
		rs1047433	T	C	0.4614	0.1804	0.1318
		rs1449077	G	T	0.5080	0.4999	0.9298
		rs1791158	T	C	0.3931	0.1938	0.3994
		rs17802774	T	C	0.9146	0.4970	0.3666
		rs57305209	G	A	0.7888	0.6770	0.7945
		rs12954678	T	C	0.5653	0.5609	0.9566
		rs1449080	C	A	0.8475	0.5818	0.5440
		rs1791202	G	A	0.6867	0.5030	0.8530
EPHA1	7	rs7786333	C	T	0.6797	0.4160	0.8373
		rs4283960	T	C	0.2295	0.2668	0.4215

rs62472728	T	C	0.2359	0.4160	0.0668
rs12703526	T	G	0.5481	0.7110	0.1124
rs11763230	T	C	0.2269	0.2640	0.1592
rs11767557	C	T	0.2314	0.2688	0.1615
rs11771145	G	A	0.7160	0.4348	0.7326
rs68140767	C	T	0.2773	0.2613	0.2880
rs79853540	A	G	0.4957	0.2904	0.4589
rs76944341	G	A	0.3052	0.1664	0.4962
rs76604048	A	G	0.3052	0.1664	0.4962
rs6951852	T	G	0.9703	0.3833	0.1113
rs67287524	G	T	0.3298	0.3166	0.3407
rs4725618	G	C	0.1522	0.6225	0.0470
rs4725619	A	T	0.1836	0.6580	0.0620
rs10228407	G	T	0.5730	0.5504	0.9889
rs62474771	C	T	0.7520	0.7076	0.3443
rs6966814	A	G	0.5789	0.5536	0.9663
rs62474772	T	C	0.7542	0.7189	0.3602
rs72611568	G	A	0.9255	0.7967	0.8118
rs9640385	T	C	0.1924	0.1523	0.9552
rs6945924	C	T	0.7138	0.6438	0.2657
rs10952552	A	G	0.0026	0.0018	0.5023
rs10245852	T	A	0.9780	0.9796	0.5554
rs12671578	A	G	0.7342	0.7044	0.7474
rs10241042	G	C	0.5734	0.5117	0.9612
rs4595035	T	C	0.7390	0.7078	0.7317
rs28375232	T	C	0.9503	0.9482	0.9916
rs72611570	A	C	0.1111	0.1122	0.5306
rs72611571	T	C	0.9200	0.9111	0.5954
rs1014069	T	C	0.6818	0.7267	0.5474
rs7789906	G	A	0.8679	0.8901	0.8812
rs10257939	C	T	0.7329	0.7810	0.7423
rs72611573	C	T	0.7105	0.8160	0.6368
rs12536735	T	C	0.5921	0.6093	0.7612
rs17164309	T	C	0.6920	0.6955	0.8851
rs78689898	T	C	0.7525	0.7065	0.5460
rs1404634	A	G	0.8199	0.5937	0.6520
rs73154237	G	A	0.8266	0.8367	0.8407

		rs1404636	T	G	0.8450	0.6170	0.6445
		rs10256611	A	C	0.4499	0.4916	0.5840
		rs10808027	C	T	0.2608	0.2283	0.5912
		rs76033591	G	A	0.3967	0.4096	0.3415
		rs1525107	A	G	0.6226	0.6117	0.7852
		rs73154244	A	G	0.7005	0.6337	0.8737
		rs1525110	C	T	0.6320	0.6220	0.7900
		rs2966709	A	G	0.8428	0.8172	0.9553
		rs2966704	A	G	0.7421	0.8242	0.2564
		rs7809756	A	C	0.5476	0.5893	0.4755
		rs55664336	C	T	0.4767	0.4351	0.4271
		rs940700	T	C	0.6767	0.9424	0.2878
		rs55716333	T	C	0.5917	0.5461	0.4696
		rs2949754	A	G	0.6486	0.9526	0.2605
		rs2966699	A	C	0.2836	0.2858	0.7656
		rs2949763	T	C	0.8964	0.8913	0.9769
		rs2949766	C	T	0.8234	0.5689	0.3179
		rs2949769	A	G	0.8916	0.9708	0.5604
		rs74726243	A	G	0.3755	0.3920	0.2031
		rs2949770	C	A	0.3038	0.3113	0.6279
FERMT2	14	rs55685925	A	G	0.8396	0.8996	0.5179
		rs4450307	G	A	0.9485	0.9743	0.4526
		rs7158114	C	G	0.7442	0.5738	0.4443
		rs8019279	T	C	0.7879	0.6125	0.4227
		rs9888615	T	C	0.5807	0.4232	0.4997
		rs7152918	A	G	0.4287	0.4454	0.1419
		rs8009633	C	G	0.3988	0.4245	0.5903
		rs74825460	T	C	0.0986	0.1087	0.4077
		rs4901317	C	T	0.0041	0.0047	0.0155
		rs957190	C	T	0.8941	0.7018	0.4352
		rs17125924	G	A	0.0849	0.0951	0.3777
		rs12878516	A	C	0.3826	0.1792	0.2808
		rs117696114	T	G	0.1039	0.1160	0.0838
		rs17125944	C	T	0.1753	0.2302	0.1423
		rs12888114	T	C	0.2836	0.2361	0.6607
		rs1958906	A	C	0.6242	0.4633	0.5009

HLA-DRB1	6	rs35067512	G	A	0.0838	0.0872	0.1492
		rs72850287	C	A	NA	NA	0.3377
		rs9270493	C	T	0.4879	0.4748	0.8649
HLA-DRB4	6	rs3828831	C	T	0.9696	0.8324	0.7488
INPP5D	2	rs12994169	A	G	0.8427	0.9901	0.5827
		rs4973063	G	A	0.6917	0.8300	0.5602
		rs4257390	T	C	0.0697	0.0696	0.7390
		rs4073363	G	A	0.9039	0.9340	0.1884
		rs6722679	A	G	0.9617	0.9544	0.9912
		rs4973065	A	G	0.3445	0.3036	0.7407
		rs56065581	T	C	0.7843	0.7956	0.6702
		rs4586623	C	T	0.3140	0.4234	0.3676
		rs7425956	T	C	0.8335	0.7168	0.3079
		rs13387054	A	G	0.9232	0.6433	0.4705
		rs4356648	C	A	0.9761	0.8797	0.3446
		rs7608422	A	G	0.5106	0.8396	0.2605
		rs7566856	G	A	0.8810	0.9471	0.5870
		rs4335931	T	C	0.2940	0.3394	0.3554
		rs4973069	C	A	0.6399	0.6168	0.8559
		rs9288684	T	C	0.3876	0.3330	0.6459
		rs9752615	A	G	0.3742	0.3116	0.5350
		rs7570061	A	G	0.5184	0.4105	0.1952
		rs68147208	G	A	0.3640	0.2910	0.2510
		rs10933432	C	G	0.0774	0.0902	0.2355
		rs10193128	C	T	0.9990	0.9872	0.9717
		rs10933435	A	G	0.5244	0.4951	0.7324
		rs10933436	C	A	0.5244	0.4951	0.7324
		rs11693862	A	G	0.5244	0.4951	0.7324
		rs11690230	T	G	0.2565	0.1974	0.8852
		rs7421653	G	A	0.2301	0.1840	0.9987
		rs7419666	C	T	0.4627	0.4466	0.9329
		rs4430948	G	A	0.8349	0.8155	0.9317
		rs6715810	T	C	0.7721	0.6023	0.2460
		rs10182994	A	G	0.4631	0.4237	0.7439

		rs12474023	A	G	0.9844	0.9863	0.6315
		rs6740918	A	G	0.1764	0.2866	0.1960
		rs3890760	C	T	0.9107	0.8918	0.9810
		rs10203185	T	C	0.6574	0.6377	0.8431
		rs1135727	A	G	0.5841	0.4912	0.5467
		rs14243	G	A	0.6524	0.6895	0.7518
MEF2C	5	rs588282	G	T	0.8110	0.5197	0.5962
		rs16903320	C	T	0.4235	0.4923	0.4838
		rs661311	T	C	0.7153	0.7014	0.2746
		rs616391	C	T	0.6917	0.4113	0.6303
		rs2247885	T	C	0.9221	0.6845	0.6307
		rs674747	G	T	0.3258	0.3360	0.4535
		rs770189	G	C	0.3558	0.1658	0.7691
		rs4521516	C	G	0.1392	0.1193	0.6678
		rs700588	A	G	0.3911	0.2160	0.6217
		rs160044	C	T	0.3739	0.2202	0.7105
		rs186233	A	G	0.2473	0.2304	0.8760
		rs302484	G	A	0.2277	0.2200	0.8105
		rs74366119	A	G	0.9492	0.9518	0.9281
		rs304145	T	A	0.1722	0.1354	0.6617
		rs216057	C	T	0.6102	0.4109	0.9957
		rs77381797	C	T	0.2938	0.2843	0.8396
		rs80043958	G	A	0.2061	0.2147	0.3315
		rs3850651	G	T	0.3263	0.2138	0.5271
		rs7737567	T	C	0.0360	0.0393	0.2992
		rs9293506	T	C	0.0081	0.0092	0.2157
		rs304141	A	G	0.7978	0.7508	0.0923
		rs74201556	C	T	0.3584	0.3752	0.4811
		rs11949307	T	G	0.0466	0.0374	0.7665
		rs770463	T	C	0.0706	0.0206	0.8429
		rs10085009	A	T	NA	NA	0.8914
		rs10075941	T	C	0.5552	0.5678	0.4374
		rs304132	A	G	0.0494	0.0705	0.1092
		rs10044186	C	T	NA	NA	0.2449
		rs190982	G	A	0.1200	0.1221	0.6341
		rs16903409	G	A	0.7270	0.8363	0.3943

		rs35372260	A	G	0.6270	0.6061	0.5780
		rs301706	G	A	0.7732	0.5258	0.1261
		rs177281	A	G	0.5395	0.5525	0.4421
		rs11952384	G	A	0.5464	0.5961	0.2889
		rs214137	A	G	0.5046	0.3462	0.4270
		rs214136	A	G	0.5042	0.3387	0.2496
		rs1455098	A	G	0.5701	0.4003	0.3886
		rs1427970	A	G	0.2001	0.1750	0.7248
		rs5869452	N	G	0.2862	0.2444	0.8548
		rs75797624	G	A	0.1841	0.1743	0.8608
MS4A4A	11	rs12283601	A	G	0.0401	0.0794	0.0495
		rs1426248	A	C	0.2599	0.3660	0.0854
		rs3758866	T	C	0.5435	0.5532	0.7294
		rs2017549	A	G	0.2668	0.5117	0.1867
		rs7104122	C	G	0.3938	0.5143	0.0402
		rs10792260	A	G	0.7062	0.6317	0.9049
		rs1365246	C	T	0.5189	0.6639	0.0368
		rs17611079	C	T	0.1396	0.1365	0.5687
		rs56157839	T	C	0.5066	0.5147	0.7357
		rs1947360	G	A	0.7471	0.7477	0.8389
		rs10750931	G	A	0.8583	0.8605	0.9231
		rs2044981	C	T	0.7028	0.4701	0.6811
		rs6591561	G	A	0.6877	0.8566	0.5309
		rs6591562	A	G	0.4730	0.6105	0.0891
		rs78926217	T	C	0.5263	0.6584	0.1071
MS4A4E	11	rs668287	T	G	0.7831	0.7396	0.3172
		rs10897016	C	T	0.1647	0.1821	0.1133
		rs11230193	C	T	0.1644	0.1811	0.1217
		rs607639	A	G	0.0318	0.0461	0.0263
		rs4939320	C	T	0.0612	0.1484	0.0419
		rs11230195	T	C	0.1418	0.1955	0.0581
		rs76174656	C	T	0.2521	0.2649	0.4887
		rs11230203	A	G	0.1647	0.1821	0.1133
		rs80340466	C	T	0.1534	0.1676	0.1508
		rs662674	A	G	0.0544	0.0734	0.0439

		rs718376	A	G	0.0562	0.1567	0.0268
		rs611267	A	G	0.6691	0.7741	0.2634
		rs650853	T	C	0.0464	0.0657	0.0309
		rs1834554	C	G	0.3028	0.2647	0.1502
MS4A6E	11	rs2304934	G	A	0.5932	0.8614	0.1462
		rs7931525	G	A	0.5750	0.9454	0.3226
		rs2289614	C	G	0.3481	0.3989	0.0399
		rs2165832	A	G	0.1388	0.2531	0.1906
		rs2289612	A	C	0.0571	0.2365	0.0128
		rs11230281	C	A	0.2208	0.0448	0.9815
		rs10736703	C	T	0.8067	0.8247	0.4895
		rs10212	C	T	0.4073	0.4861	0.4796
		rs2289610	C	T	0.0488	0.0712	0.1295
NME8	7	rs4723713	A	G	0.6531	0.5702	0.8799
		rs921397	T	C	0.7973	0.7902	0.7353
		rs4720262	T	C	0.6418	0.5823	0.2743
		rs2598046	G	A	0.5168	0.4949	0.6983
		rs2722372	A	G	0.9992	0.8145	0.1777
		rs59301349	G	A	0.9830	0.9995	0.5883
		rs55940848	A	G	0.7287	0.5864	0.8702
		rs10270230	A	C	0.6681	0.9605	0.4927
		rs2722353	C	A	0.8729	0.9530	0.1811
		rs2722355	A	G	0.7234	0.6871	0.4993
		rs77524454	A	G	0.9574	0.9084	0.1805
		rs17171184	A	G	0.9047	0.8722	0.5496
		rs2249451	G	A	0.8611	0.5426	0.2526
		rs2290224	G	A	0.7567	0.6346	0.9580
		rs118137117	T	C	0.9539	0.9688	0.2151
		rs3807171	C	T	0.7826	0.7040	0.7095
		rs1450845	G	A	0.8321	0.8128	0.8897
		rs2722311	A	G	0.6610	0.6210	0.9980
		rs2598031	G	A	0.7584	0.7177	0.9666
		rs77113930	A	G	0.9566	0.9759	0.7189
		rs62001868	G	A	0.6127	0.5697	0.9050
		rs2598021	A	G	0.8152	0.9121	0.5539

		rs2598017	C	G	0.2340	0.0993	0.9313
		rs2598015	G	A	0.6147	0.4999	0.5644
		rs12532321	A	G	0.7571	0.7220	0.5769
		rs75991941	T	C	0.9122	0.8705	0.4769
		rs935765	T	C	0.6062	0.4839	0.5275
		rs2598010	T	C	0.6022	0.5014	0.6235
		rs2722366	C	A	0.6883	0.3769	0.6670
		rs6462793	C	G	0.8726	0.9112	0.4289
		rs2722365	G	A	0.5361	0.2858	0.5333
		rs1839585	A	C	0.8642	0.9834	0.3348
		rs10268173	G	T	0.8507	0.6296	0.5153
		rs2722349	C	A	0.3069	0.2790	0.6515
		rs2598006	G	C	0.6887	0.6872	0.9533
		rs2598124	G	A	0.5920	0.5974	0.8076
		rs10488616	G	T	0.9093	0.7372	0.7153
		rs2598123	C	A	0.7316	0.7172	0.9081
		rs3807184	C	G	0.9954	0.9746	0.6349
		rs3807185	T	C	0.6980	0.6839	0.6568
		rs7807193	G	A	0.9160	0.9729	0.7409
		rs113865274	T	G	0.8690	0.8948	0.7833
		rs2242026	T	C	0.7473	0.7670	0.8082
		rs72638248	C	T	0.4658	0.4476	0.5550
		rs71546608	A	G	0.2120	0.2106	0.6869
PICALM	11	rs592314	A	G	0.0426	0.0187	0.9974
		rs11234495	T	C	0.1150	0.6105	0.0391
		rs510566	T	G	0.4308	0.4348	0.7060
		rs17209625	T	A	0.4638	0.3570	0.3182
		rs17817201	T	C	0.3133	0.3047	0.9107
		rs35206999	C	T	0.7605	0.7654	0.7994
		rs642949	A	G	0.1402	0.0841	0.7560
		rs664629	G	A	0.2972	0.3178	0.4813
		rs17817931	C	T	0.7558	0.7598	0.8227
		rs669556	C	T	0.0473	0.0261	0.8591
PLD3	19	rs4490097	A	C	0.0828	0.3329	0.0488
		rs7507651	T	G	0.0600	0.3177	0.0278

		rs12151243	T	G	0.8260	0.8112	0.8841
		rs11083554	G	C	0.3246	0.9739	0.0768
		rs11672825	T	G	0.0160	0.1928	0.0071
PTK2B	8	rs6986075	C	T	0.9143	0.7522	0.4019
		rs6557991	T	C	0.3678	0.3144	0.6825
		rs4291226	A	G	0.6822	0.6880	0.8086
		rs2322719	A	G	0.7464	0.6819	0.5396
		rs117601945	A	C	0.8368	0.8154	0.6248
		rs74317051	T	C	0.8885	0.8969	0.7126
		rs28547290	G	A	0.8892	0.3996	0.3074
		rs1879189	A	G	0.8346	0.4673	0.4221
		rs76082706	A	G	0.2631	0.2568	0.3585
		rs4534095	G	A	0.4106	0.4078	0.8593
		rs6987305	A	G	0.6258	0.6905	0.5866
		rs10109834	C	A	0.0372	0.0346	0.7298
		rs7016131	T	C	0.6617	0.3100	0.5403
		rs57729364	A	C	NA	NA	0.9514
		rs10097651	T	C	0.3158	0.3266	0.4095
		rs2322609	G	A	0.7498	0.7509	0.8689
		rs13266887	C	T	0.3448	0.3510	0.6748
		rs6557994	C	T	0.7118	0.7189	0.8472
		rs1429938	G	A	0.8281	0.7103	0.3555
		rs1429937	T	C	0.8020	0.7631	0.4590
		rs4732720	A	G	0.7524	0.6619	0.4991
		rs10086852	C	G	0.5193	0.4114	0.3377
		rs17057065	T	C	0.6839	0.7809	0.2882
		rs11776858	G	A	0.5121	0.5911	0.3795
		rs11777664	G	A	0.5101	0.5982	0.3415
		rs919491	A	G	0.5195	0.5950	0.3979
		rs919492	G	T	0.5195	0.5950	0.3979
		rs939267	T	C	0.7242	0.8633	0.4502
		rs17447958	T	C	0.7746	0.6741	0.4387
		rs1128433	G	A	0.5195	0.5950	0.3979
		rs1045511	A	G	0.4581	0.5237	0.3976
		rs1045512	G	A	0.5195	0.5950	0.3979
		rs2322718	G	T	0.5195	0.5950	0.3979

		rs2115805	A	G	0.5195	0.5950	0.3979
		rs2059968	T	A	0.5195	0.5950	0.3979
		rs2059969	C	A	0.5195	0.5950	0.3979
		rs7005183	G	A	0.7091	0.7695	0.6691
		rs1367088	T	C	0.3407	0.9092	0.0516
		rs17376459	G	A	0.8487	0.8306	0.4498
		rs7000615	G	T	0.9627	0.8072	0.8573
		rs3779633	A	C	0.8862	0.8166	0.8059
		rs725788	C	T	0.3391	0.2545	0.4635
		rs725787	G	A	0.8534	0.8354	0.4589
		rs3757908	T	C	0.9621	0.6988	0.7475
		rs4733057	G	A	0.8061	0.5098	0.7874
		rs2082100	T	C	0.9208	0.8572	0.7130
		rs17377462	A	G	0.8685	0.8126	0.3646
		rs919493	T	C	0.8562	0.8815	0.6426
		rs11995441	C	T	0.3917	0.2755	0.1043
		rs9773817	G	A	NA	NA	0.1630
		rs9657295	A	G	0.7317	0.9334	0.2341
		rs11135993	C	T	0.9486	0.8456	0.2743
		rs1019832	A	G	0.7773	0.7218	0.5786
		rs7846626	C	G	0.3497	0.2463	0.1455
RIN3	14	rs7141239	T	C	0.4298	0.8192	0.1868
		rs1952330	T	C	0.6357	0.6735	0.6246
		rs7160605	C	A	0.7616	0.7909	0.7342
		rs56046485	G	A	0.1935	0.1785	0.6662
		rs34071903	C	G	0.6854	0.6932	0.8442
		rs7157447	C	T	0.9161	0.8611	0.7586
		rs10141468	T	C	0.4224	0.6483	0.3845
		rs8008855	T	C	0.1538	0.1376	0.8361
		rs4374096	G	A	0.1965	0.1950	0.4705
		rs10498634	G	A	0.3093	0.1610	0.7209
		rs77826962	G	A	0.7966	0.8219	0.7691
		rs10467865	G	A	0.2216	0.3673	0.0294
		rs4900135	C	T	0.2398	0.6372	0.0667
		rs1887559	C	T	0.1601	0.5709	0.0710
		rs10467867	G	A	0.1647	0.5789	0.0722

rs11846004	A	G	0.6719	0.2865	0.4317
rs12893289	A	G	0.7517	0.2477	0.2640
rs10141449	A	G	0.9294	0.9135	0.1924
rs12884739	A	G	0.2369	0.0500	0.5464
rs736087	A	G	0.4553	0.4626	0.7144
rs8008611	A	G	0.0877	0.1127	0.1582
rs2104237	C	T	0.2773	0.4035	0.1739
rs1885747	G	A	0.0097	0.0150	0.0399
rs11160075	A	G	0.2881	0.1960	0.7473
rs2402171	A	G	0.2202	0.3117	0.2433
rs2402172	C	A	0.1579	0.2747	0.0638
rs11623185	G	A	0.5145	0.5094	0.9142
rs12433867	G	A	0.8187	0.4525	0.4165
rs12435667	A	C	0.8041	0.4312	0.1801
rs8012413	A	G	0.9853	0.8283	0.0245
rs80267197	A	G	0.9882	0.9763	0.4989
rs942058	G	C	0.2567	0.4335	0.1344
rs9788510	A	C	0.0760	0.1120	0.0656
rs79113623	T	C	0.0602	0.0899	0.0569
rs4904957	A	G	0.2496	0.8781	0.0103
rs9788624	A	G	0.0768	0.1137	0.0682
rs9788457	C	T	0.3197	0.6101	0.0189
rs749647	A	C	0.8473	0.9012	0.4655
rs11160080	T	C	0.9222	0.7736	0.6208
rs61975788	T	C	0.1415	0.2629	0.0519
rs4904960	C	T	0.0329	0.0346	0.2626
rs943655	A	C	0.0313	0.0270	0.4052
rs10498635	T	C	NA	NA	0.8517
rs11627032	C	T	0.1285	0.2603	0.0349
rs4904970	T	C	0.6998	0.7138	0.4629
rs8017311	A	G	0.0166	0.0320	0.0771
rs11629324	T	C	0.7047	0.9805	0.1431
rs12885166	T	G	0.3481	0.1606	0.8894
rs9323880	T	C	0.1081	0.1738	0.0434
rs11160087	A	G	0.8066	0.9433	0.2187
rs6575272	G	A	0.5635	0.5338	0.8791
rs942063	G	A	0.6229	0.7487	0.2212

SLC24A4	14	rs72631607	A	G	0.1548	0.0321	0.9177
		rs6575246	A	G	0.1351	0.1012	0.4857
		rs2402130	G	A	0.5975	0.5892	0.4134
		rs8013152	T	C	0.4639	0.4547	0.2006
		rs56691294	G	T	0.2218	0.2794	0.1188
		rs12887287	G	A	0.1301	0.3397	0.1238
		rs35778179	T	C	0.4424	0.4687	0.2725
		rs2010747	C	T	0.4666	0.4534	0.1267
		rs66753927	A	G	0.9206	0.9360	0.7131
		rs4904886	G	A	0.4437	0.3170	0.7357
		rs12891074	G	A	0.6761	0.6547	0.9451
		rs61975636	G	A	0.8869	0.9126	0.5304
		rs12435939	T	C	0.7063	0.7058	0.9177
		rs8020526	C	T	0.3218	0.1535	0.2749
		rs8022236	A	G	0.1643	0.0436	0.1360
		rs4347559	G	A	0.9010	0.8900	0.6693
		rs59506823	A	G	0.9037	0.9298	0.7490
		rs72695142	A	G	0.5837	0.6344	0.2782
		rs4904889	T	C	0.3348	0.3434	0.6210
		rs61583500	C	T	0.2373	0.1896	0.7558
		rs4904890	A	G	0.6236	0.6811	0.2350
		rs4904896	C	T	0.2630	0.2018	0.8253
		rs4904897	T	C	0.6209	0.6847	0.1945
		rs11845652	G	A	0.9840	0.9619	0.7595
		rs4904901	T	G	0.0869	0.0858	0.4901
		rs11624865	G	A	0.0977	0.0877	0.6815
		rs4904903	A	G	0.3545	0.4218	0.0725
		rs12435024	A	G	0.0065	0.0054	0.2517
		rs17128291	G	A	0.4486	0.4829	0.3496
		rs10138927	C	G	0.0378	0.0411	0.3164
		rs10431740	T	C	0.0055	0.0041	0.2292
		rs12588761	G	A	0.4606	0.5471	0.5264
		rs28579858	T	C	0.2336	0.2288	0.9040
		rs7152962	G	A	0.1715	0.2440	0.2779
		rs10147889	G	A	0.9805	0.8315	0.2242
		rs8016437	C	T	0.9163	0.9094	0.2461

rs77185674	A	G	0.4314	0.4253	0.8522
rs12432774	A	G	0.6943	0.7049	0.6978
rs734504	A	G	0.7072	0.3103	0.1917
rs12433292	T	C	0.6607	0.7350	0.2835
rs12588868	T	C	0.7663	0.7220	0.6851
rs74073080	T	C	0.6821	0.6763	0.9684
rs61977294	A	G	0.3780	0.4437	0.0703
rs11160066	C	T	0.3739	0.8892	0.1401
rs8015178	T	C	0.3696	0.9094	0.0760
rs7144273	C	T	0.5424	0.3721	0.9329
rs10137770	G	A	0.6691	0.9799	0.1000
rs7158400	T	C	0.2803	0.2277	0.8513
rs10498633	T	G	0.1667	0.1646	0.9190
rs10134832	A	G	0.1045	0.0519	0.1452
rs61977311	A	G	0.0099	0.0256	0.0049
rs67063100	A	G	0.0447	0.0811	0.0038
rs11160069	T	C	0.0380	0.1208	0.0537
rs77248792	G	A	0.1808	0.1004	0.1499
rs9323877	G	A	0.1265	0.2512	0.1735
rs61116143	T	G	0.2851	0.2998	0.5559
rs4497611	G	C	0.0528	0.0680	0.2173
rs4904929	C	T	0.0528	0.0680	0.2173
rs11160070	C	T	0.7295	0.9838	0.3136
rs11160071	T	C	0.7521	0.8451	0.5254
rs12434016	G	T	0.0041	0.0194	0.0127
rs78476646	C	A	0.6939	0.7315	0.6029
rs1952323	G	A	0.6958	0.7409	0.5542
rs10431637	C	A	0.0998	0.1395	0.0199
rs2149054	C	A	0.7081	0.7496	0.5884
rs2182838	T	C	0.5183	0.5559	0.5021
rs12589691	A	G	0.7353	0.7320	0.8874
rs10135174	A	G	0.2216	0.2631	0.2319
rs11623937	A	G	0.3357	0.2618	0.9762
rs17783630	C	A	0.5786	0.6667	0.5665
rs10150603	T	C	0.2217	0.2618	0.2419
rs61977322	C	T	0.8523	0.8373	0.6144
rs7150406	A	G	0.4167	0.4277	0.6682

		rs79685763	A	G	0.4541	0.3118	0.6883
		rs78739077	T	G	0.0253	0.0287	0.1273
		rs76960688	C	T	0.1290	0.1436	0.0905
SORL1	11	rs4935774	C	T	0.3497	0.3645	0.5922
		rs578506	G	C	0.8413	0.8498	0.2665
		rs3824963	G	A	0.2871	0.4009	0.0558
		rs661057	T	C	0.8823	0.1551	0.0373
		rs3862605	C	T	0.2521	0.3450	0.0460
		rs3862606	G	A	0.4101	0.2867	0.7502
		rs3781825	C	T	0.5769	0.4597	0.0163
		rs4936632	G	A	0.6016	0.3854	0.0330
		rs11218301	A	G	0.9097	0.9743	0.2599
		rs3781826	A	T	0.7921	0.6987	0.3020
		rs33961761	N	G	0.4740	0.2148	0.7949
		rs11218304	G	A	0.7490	0.8138	0.2323
		rs12276905	G	A	0.7811	0.6777	0.1648
		rs4631890	A	G	0.1456	0.0441	0.2666
		rs985421	A	G	0.8347	0.6742	0.1485
		rs687228	A	G	0.7619	0.3440	0.0600
		rs987482	C	T	0.8897	0.8136	0.1136
		rs668387	C	T	0.8280	0.2868	0.0693
		rs200748970	A	G	NA	NA	0.9911
		rs2298813	A	G	0.6364	0.6425	0.8014
		rs11218319	T	C	0.3480	0.3781	0.2164
		rs666004	G	A	0.0942	0.1189	0.2287
		rs11501162	T	C	0.1441	0.1452	0.6954
		rs3781827	G	A	0.9796	0.3514	0.2783
		rs10892754	G	A	0.8711	0.3118	0.3557
		rs79187659	T	C	0.8823	0.9194	0.2858
		rs2276345	C	A	0.5255	0.7619	0.0515
		rs11218325	C	A	0.9186	0.4288	0.0861
		rs11604897	T	C	0.0652	0.0732	0.3276
		rs7946599	A	G	0.8875	0.8746	0.7415
		rs6589885	A	G	0.8876	0.8746	0.7376
		rs556349	G	T	0.9843	0.8308	0.1729
		rs11218339	G	A	0.6422	0.6401	0.9547

		rs1699108	T	A	0.8639	0.7944	0.1654
		rs1784920	A	G	0.3009	0.3454	0.4484
		rs1790213	G	A	0.6614	0.7976	0.5237
		rs4420280	C	A	0.7323	0.5538	0.0872
		rs67140863	C	G	0.6423	0.4525	0.0591
		rs117806822	T	C	0.1852	0.1759	0.9337
		rs2070045	T	G	0.9955	0.8895	0.8112
		rs3781835	A	G	0.4568	0.4370	0.3541
		rs59260691	A	G	0.0513	0.0503	0.4766
		rs1625828	A	G	0.7936	0.9453	0.5156
		rs10892759	G	A	0.7339	0.9530	0.3543
		rs1620003	A	G	0.4345	0.3996	0.2779
		rs1629493	A	G	0.7208	0.6253	0.1734
		rs3737529	T	C	0.6981	0.8582	0.2200
		rs726601	C	T	0.7724	0.8929	0.6683
		rs1784933	G	A	0.4917	0.4646	0.8493
		rs10892761	T	C	0.7491	0.5717	0.8318
		rs117097913	G	A	0.3336	0.3247	0.2718
ZCWPW1	7	rs2286266	T	G	0.0701	0.0682	0.8958
		rs3815257	A	G	0.5008	0.5577	0.4795
		rs2286265	T	C	0.2052	0.1669	0.3327
		rs5015755	T	G	0.0352	0.0321	0.7478
		rs5015756	C	T	0.4979	0.4107	0.8601

A1 = minor allele, A2 = major allele, AD = Alzheimer's disease, Chr = chromosome, MMSE = Mini-Mental State Examination.

Analysis was obtained after adjustment for covariates including age, gender, and education.

Supplementary Table S2. (A) The distribution of *APOE* alleles; (B) Results of analyses of association between the MMSE scores and the *APOE* ε4 allele.

A.

Gene	CHR	ε2 (%)	ε3 (%)	ε4 (%)
APOE	19	6.2	82.7	11.1

B.

Analysis	BETA	SE	P
(1) *APOE* ε4 (carriers versus non-carriers)			
	-0.02	0.04	0.8469
(2) *APOE* ε4 (heterozygose versus non-carriers)			
	-0.02	0.04	0.7478
(3) *APOE* ε4 (homozygose versus non-carriers)			
	-0.02	0.04	0.6713

BETA = Beta coefficients, Chr = chromosome, MMSE = Mini-Mental State Examination, SE = standard error.

Analysis was obtained after adjustment for covariates including age, gender, and education.

Supplementary Table S3. Multivariable logistic regression analysis for the two-way gene-gene interaction models.

Two-way interaction model	OR	95% CI	P value[b]
(a) *CASS4* and *SLC24A4*			
rs911159 (GG genotype) with rs67063100 (GG genotype)[a]	1		
rs911159 (AA genotype) with rs67063100 (GG genotype)	7.05	1.19-41.77	**0.0315**
rs911159 (AG genotype) with rs67063100 (AA+AG genotype)	2.25	1.04-4.89	**0.0399**
rs911159 (AG genotype) with rs67063100 (GG genotype)	1.34	0.71-2.54	0.3708
rs911159 (GG genotype) with rs67063100 (AA+AG genotype)	1.87	1.05-3.31	**0.0328**
(b) *EPHA1* and *SLC24A4*			
rs10952552 (GG genotype) with rs67063100 (GG genotype)[a]	1		
rs10952552 (AA genotype) with rs67063100 (GG genotype)	0.38	0.08-1.74	0.2152
rs10952552 (AG genotype) with rs67063100 (AA+AG genotype)	2.26	1.12-4.58	**0.0232**
rs10952552 (AG genotype) with rs67063100 (GG genotype)	0.95	0.51-1.77	0.8799
rs10952552 (GG genotype) with rs67063100 (AA+AG genotype)	1.49	0.81-2.72	0.1994

(c) *FERMT2* and *MEF2C*			
rs4901317 (TT genotype) with rs9293506 (CC genotype)[a]	1		
rs4901317 (CC genotype) with rs9293506 (CC genotype)	5.56	0.48-64.75	0.1711
rs4901317 (CT genotype) with rs9293506 (TC genotype)	2.13	0.42-10.72	0.3582
rs4901317 (CT genotype) with rs9293506 (CC genotype)	1.67	0.75-3.72	0.2065
rs4901317 (TT genotype) with rs9293506 (TC genotype)	1.67	0.97-2.89	0.0653
rs4901317 (TT genotype) with rs9293506 (TT genotype)	5.17	0.86-31.16	0.0733
(d) *FERMT2* and *SLC24A4*			
rs4901317 (CT genotype) with rs67063100 (AA+AG genotype)[a]	1		
rs4901317 (TT genotype) with rs67063100 (AA+AG genotype)	0.37	0.13-1.06	0.0652
rs4901317 (CC genotype) with rs67063100 (GG genotype)	1.29	0.09-17.95	0.8516
rs4901317 (CT genotype) with rs67063100 (GG genotype)	0.22	0.06-0.89	**0.0334**
rs4901317 (TT genotype) with rs67063100 (GG genotype)	0.23	0.08-0.64	**0.0047**
(e) *MEF2C* and *SLC24A4*			

rs9293506 (CC genotype) with rs67063100 (GG genotype)[a]	1		
rs9293506 (CC genotype) with rs67063100 (AA+AG genotype)	1.86	1.07-3.24	**0.0275**
rs9293506 (TC genotype) with rs67063100 (AA+AG genotype)	2.79	1.17-6.68	**0.0210**
rs9293506 (TC genotype) with rs67063100 (GG genotype)	1.67	0.88-3.17	0.1134
rs9293506 (TT genotype) with rs67063100 (AA+AG genotype)	4.60	0.25-83.37	0.3021
rs9293506 (TT genotype) with rs67063100 (GG genotype)	7.01	0.70-69.95	0.0970

CI = confidence interval, OR = odds ratio.

[a] Reference.

[b] Versus reference. Analysis was obtained after adjustment for covariates including age, gender, and education. P values of < 0.05 are shown in bold.

Supplementary Table S4. Imputed SNPs in 2 AD-related genes.

Gene	CHR	SNP	A1	A2	MAF
CASS4	20	rs7274581	C	T	0.00
		rs16979934	C	T	0.00
		rs6024870	A	G	0.00
PLD3	19	rs145999145	A	G	0.00

A1 = minor allele, A2 = major allele, AD = Alzheimer's disease, Chr = chromosome,

MAF = minor allele frequency.
